# Measured weight loss as a precursor to cancer diagnosis: retrospective cohort analysis of 43 302 primary care patients

**DOI:** 10.1002/jcsm.13051

**Published:** 2022-07-28

**Authors:** Brian David Nicholson, Matthew James Thompson, Frederick David Richard Hobbs, Matthew Nguyen, Julie McLellan, Beverly Green, Jessica Chubak, Jason Lee Oke

**Affiliations:** ^1^ Nuffield Department of Primary Care Health Sciences University of Oxford Oxford UK; ^2^ Department of Family Medicine University of Washington Seattle WA USA; ^3^ Kaiser Permanente Washington Health Research Institute Seattle WA USA

**Keywords:** Weight loss, Cancer diagnosis, Electronic health records, Primary care

## Abstract

**Background:**

Unexpected weight loss is a presenting feature of cancer in primary care. Data from primary care are lacking to quantify how much weight loss over what period should trigger further investigation for cancer. This research aimed to quantify cancer diagnosis rates associated with measured weight change in people attending primary care.

**Methods:**

Retrospective cohort study of primary care electronic health records data linked to the Surveillance, Epidemiology, and End Results cancer registry (Integrated healthcare delivery system in Washington State, United States). Multivariable Cox regression incorporating time varying covariates using splines to model non‐linear associations (age, percentage weight change, and weight change interval). Fifty thousand randomly selected patients aged 40 years and over followed for up to 9 years (1 January 2006 to 31 December 2014). Outcome measures are hazard ratios (95% confidence intervals) to quantify the association between percentage weight change and cancer diagnosis for all cancers combined, individual cancer sites and stages; percentage risk of cancer diagnosis within 6 months of the end of each weight change episode; and the positive predictive value for cancer diagnosis.

**Results:**

There were 43 302 included in the analysis after exclusions. Over 287 858 patient‐years of follow‐up, including 24 272 (56.1%) females, 23 980 (55.4%) aged 40 to 59 years, 15 113 (34.9%) 60 to 79 years, and 4209 (9.7%) aged 80 years and over. Adjusted hazard ratios (95% confidence interval) for cancer diagnosis in a 60 years old ranged from 1.04 (1.02 to 1.05, *P* < 0.001) for 1% weight loss to 1.44 (1.23 to 1.68, *P* < 0.001) for 10%. An independent linear association was observed between percentage weight loss and increasing cancer risk. The absolute risk of cancer diagnosis increased with increasing age (up to 85 years) and as the weight change measurement interval decreased (<1 year). The positive predictive value for a cancer diagnosis within 1 year of ≥5% measured weight loss in a 60 to 69 years old was 3.41% (1.57% to 6.37%) in men and 3.47% (1.68% to 6.29%) in women. The risk of cancer diagnosis was significantly increased for pancreatic, myeloma, gastro‐oesophageal, colorectal, breast, stage II and IV cancers.

**Conclusions:**

Weight loss is a sign of undiagnosed cancer regardless of the interval over which it occurs. Guidelines should resist giving an arbitrary cut‐off for the interval of weight loss and focus on the percentage of weight loss and the patient's age. Future studies should focus on the association between diagnostic evaluation of weight change and risk of cancer mortality.

## Introduction

Most cancers are diagnosed after individuals present to clinical settings with symptoms and signs of cancer, rather than after routinely recommended screening.^1^, ^2^, ^3^ Improving the range of cancers detectable by screening and optimizing uptake of recommended screening services are important for reducing cancer burden.^4^, ^5^ However, it is also critical to understand how individuals with cancer present in clinical settings since not all cancers will be detected by screening, especially in an early stage when cancer is more treatable.^6^, ^7^ This is particularly important for cancers where diagnosis at a late stage is common, and therapeutic options are limited, such as cancers of the lung, pancreas, and ovary.^8^, ^9^

While some patients present to primary care settings with clinical features that are strongly associated with specific cancers, many present with non‐specific features.^10^ Unexpected weight loss (UWL) has been identified as a non‐specific presenting feature of cancer in primary care settings, where it may provide an early sign of cancer.^11^, ^12^, ^13^, ^14^ UWL may go undetected or may be misattributed due to diurnal fluctuations in weight, expected weight changes with age, diet or exercise, obesity, and variation in weight measurement frequency.^14^, ^15^ As most patients presenting to primary care with UWL will not have cancer, diagnostic strategies that avoid the harms of unnecessary invasive and costly investigation are required.^13^, ^16^, ^17^, ^18^, ^19^

The amount of weight loss and the period of time over which it occurs that is most strongly associated with underlying cancer remains poorly defined.^12^, ^15^, ^20^ Almost all studies in this area are retrospective observational studies that define weight loss by means of diagnostic codes entered into the electronic health record (EHR).^12^ However, these codes are based on primary care providers' (PCPs') decisions that the degree of weight loss is sufficiently concerning to justify recording it. Given that weight is measured routinely at primary care visits in the USA, there is likely to be sufficiently complete data to allow detailed analysis of weight change over time and associations with incident cancers.^21^ The percentage loss of weight is likely to be most clinically relevant, as change relative to baseline weight is more meaningful than the absolute change.

We aimed to investigate whether measured weight loss was independently associated with an increased risk of subsequent cancer diagnosis and to investigate the association between the amount and period of weight loss and cancer risk using primary care EHR data from the USA.

## Methods

The study population comprised 50 000 randomly selected patients aged ≥40 years with a Kaiser Permanente Washington (KPWA) primary care provider followed for up to 9 years between 01/01/2006 and 31/12/2014; 50 000 was the maximum number of patients authorized by KPWA. Access to the database and creation of the study population was conducted solely by one author (M. N.) at KPWA. Data cleaning, prior to creation of the study population, involved dropping patients when the weight values were null or determined to be incorrect (<20 kg, >245 kg).

### Setting

KPWA provides health care and coverage to enrolled members, including those with government (e.g. Medicare) insurance, employer‐based insurance, and private insurance. About two thirds of members receive primary care from KPWA providers at one of the system's own medical centres; however, many of them receive speciality care at externally owned facilities. Most of KPWA's members reside within western Washington State, which is within the catchment area of a Surveillance, Epidemiology, and End Results (SEER) cancer registry. The KPWA database is routinely updated from SEER, the linkage is based on medical record number, social security number, last name, gender, and date of birth.

### Inclusions and exclusions

**Figure 1:**
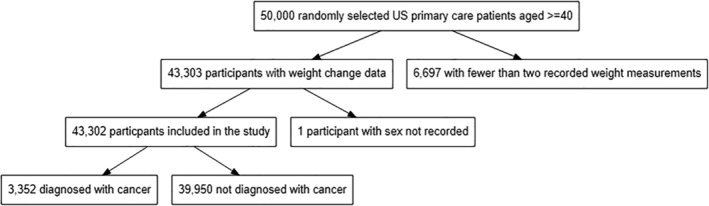
Flow diagram of patients.

Participants entered the cohort on their birthday in 2006 if they were aged ≥40 years, and resided in the local cancer registry catchment area. Follow‐up data were collected until the earliest of: disenrollment from KPWA, relocation out of the registry catchment area, death, a first code indicating bariatric surgery, or the patient's birthday in 2014. Patients were excluded if they had fewer than two weight measurements recorded in the EHR during the study period or if they had missing data on sex (*Figure* 1).

### Weight change episodes

A weight change episode was defined as the time between two weight measurements. For each episode, the interval of weight change was defined as the difference in time between the two weight measurements, and percentage weight change was calculated between the two weight measurements (Supporting Information, *Figure* S1).

### Cancer diagnoses

Incident cancer diagnoses during the study period were identified from routine linkages between KPWA and the local SEER cancer registry using ICD‐O‐3 coding and grouped according to anatomical location and common investigative approach, for example gastric and oesophageal were combined as gastro‐oesophageal (Supporting Information, *Table* S1). Registry data included date of diagnosis, tumour location, and stage. Non‐melanoma skin cancers were excluded.

### Statistical analysis

The association between measured weight loss and the rate of cancer diagnosis was estimated using multivariable Cox regression. Patients were censored at the earliest of date of cancer diagnosis and the end of follow‐up. As covariate status changed throughout the study period, time‐varying covariates were included for all covariates except baseline weight and previous cancer diagnosis. Patients could contribute multiple weight change episodes if they had more than two weight measurements in the study period; however, we examined weight change only between consecutive measurements. Non‐linear associations were investigated between continuous covariates (age, percentage weight change, and weight change interval) using smoothing splines.

Minimally adjusted models were fitted including baseline weight at cohort entry (centred at 80 kgs), percentage weight change, age (centred at 60 years), sex, weight change interval, and tobacco use. Fully adjusted models also included ethnicity, previous cancer diagnosis, and co‐morbidities associated with weight loss in the literature (chronic heart failure, chronic obstructive pulmonary disease, dementia, depression, inflammatory bowel disease, renal failure, rheumatoid arthritis, and thyroid disease). The date of the first record of each co‐morbidity changed the indicator status from absent to present in the analysis from that time point onward. The purpose of fitting both minimally and fully adjusted models was to minimize data loss and to assess whether the association between weight loss and cancer was sensitive to model specification.

We assessed effect modification by assessing two‐way interaction terms between percentage weight change and age at beginning of episode, sex, baseline weight, and weight change interval. The proportional hazards assumption was assessed for each model using weighted residuals as proposed by Grambsch and Therneau (cox.zph() in R) and by checking plots of the Schoenfeld residuals. Hazard ratios (HR), 95% confidence intervals (95% CI), and *P*‐values were calculated to quantify the association between percentage weight change and the rate of cancer diagnosis for all cancers combined, for individual cancer site, and for cancer stage.

The percentage risk of cancer diagnosis within 6 months of the end of each weight change episode was predicted using the fully adjusted model for the categories of percentage weight change (0%, 2.5%, 5%, 7.5%, and 10%), interval of weight measurement (1, 3, 6, and 12 months) and age by year (41–90 years). These were presented as line plots for men and women.

The positive predictive value (PPV) for a cancer diagnosis within 6 and 12 months after ≥5% weight loss was calculated by randomly sampling one weight change episode per patient from the dataset, stratified by age‐group at start of episode (40–49 years, 50–59 years, 60–69 years, 70–79 years, and ≥80 years), and sex.

### Sensitivity analyses

Four sensitivity analyses were conducted. We re‐ran the analysis excluding people with a record of past cancer to assess whether a previous cancer diagnosis confounded the relationship between weight loss and cancer risk. We controlled for the frequency of consultations including a weight measurement by adding a term for consultation frequency in the 90 day period up until the current weight measurement as more frequent attendance to primary care has been previously associated with an increased risk of a cancer diagnosis.^22^ We randomly sampled a single episode per patient and refitted the minimally adjusted models to remove the potential for bias associated with incorporating multiple measurements per patient. Finally, we included codes for other symptoms to identify whether the association between weight change and cancer diagnosis remained after taking other symptoms into account.

For the selection of splines a *P* < 0.01 was used. For all other analyses, statistical significance was defined as *P* < 0.05. Clinical significance was defined as a 50% increase in the rate of cancer. All analyses were conducted in R version 4.0.2.

### Patient and public involvement

Patients and the public were involved in an advisory capacity in the application for funding to support this research. An advisory panel of patient and public members provided comments on a related article that informed the current analysis. Patients were not directly involved in the conduct or analysis of the study. The results of this study will be disseminated through the media channels of the host institution of the lead author and the funder for scientific and lay audiences.

## Results

After excluding 6697 people with fewer than two recorded weight measurements, and one with no sex recorded, 43 302 patients were included in the analysis with over 287 858 person‐years of follow‐up (6.65 years per patient on average). There were 3352 (7.79%) cancers diagnosed during the study period, 19 030 (43.9%) people were male (17 491 non‐cancer, 1539 cancer), 25 130 (58.02%) aged 40 to <60 years (22 934 non‐cancer, 1046 cancer), and 32 638 (75.4%) white (29 682 non‐cancer, 2956 cancer). Of 830 960 weight measurements, 830 329 (99.93%) were recorded at ambulatory care visits, 611 (0.07%) at emergency department visits, and 20 (0.002%) at other encounters. The median (IQR) number of weight change episodes per person was 13^6^, ^23^ at a median (IQR) interval of 70.5 (38 143.5) days (*Table* 1). The most common cancers were breast (612, 18.26%), prostate (360, 10.74%), and lung (348, 10.38%).

**Table 1:** Cohort characteristics of patients with at least 2 weight measurements during the period 2006–2014, overall and by cancer diagnosis during follow‐up (*N* = 43 302)

Variables	Category/Statistic	Overall, *n* (%)	No cancer, *n* (%)	Cancer, *n* (%)
*At cohort entry*				
Sex	Female	24 272 (56.1)	22 459 (56.2)	1813 (54.1)
	Male	19 030 (43.9)	17 491 (43.8)	1539 (45.9)
Age (years)	40 to 59	23 980 (55.4)	22 934 (57.4)	1046 (31.2)
	60 to 79	15 113 (34.9)	13 353 (33.4)	1760 (52.5)
	80+	4209 (9.7)	3663 (9.2)	546 (16.3)
Ethnicity	White	32 638 (75.4)	29 682 (74.3)	2956 (88.2)
	Asian	2878 (6.6)	2700 (6.8)	178 (5.3)
	Black	1698 (3.9)	1589 (4.0)	109 (3.3)
	Native Hawaiian/Pacific Islander	216 (0.5)	206 (0.5)	10 (0.3)
	Mixed	856 (2.0)	815 (2.0)	41 (1.2)
	Native American	308 (0.7)	296 (0.7)	12 (0.4)
	Other	644 (1.5)	605 (1.5)	39 (1.2)
	Missing	4064 (9.4)	4057 (10.2)	7 (0.2)
Past cancer	Yes	4112 (9.5)	3552 (8.9)	560 (16.7)
Smoking	Never	7778 (18.0)	7263 (18.2)	515 (15.4)
	Current	1986 (4.6)	1827 (4.6)	159 (4.7)
	Passive	100 (0.2)	97 (0.2)	3 (0.1)
	Former	4987 (11.5)	4534 (11.3)	453 (13.5)
	Missing	28 451 (65.7)	26 229 (65.7)	2222 (66.3)
Weight (kg)	Mean (SD)	83.48 (21.47)	83.50 (21.59)	83.22 (19.95)
Body mass index (kg/m^2^)	Under 20	853 (2.0)	801 (2.0)	52 (1.6)
	20 to 24	7952 (18.4)	7308 (18.3)	644 (19.2)
	25 to 29	12 444 (28.7)	11 373 (28.5)	1071 (32.0)
	30 to 34	8218 (19.0)	7565 (18.9)	653 (19.5)
	35 to 39	3808 (8.8)	3515 (8.8)	293 (8.7)
	40 to 50	2342 (5.4)	2170 (5.4)	172 (5.1)
	Missing	7685 (17.7)	7218 (18.1)	467 (13.9)
Adrenal insufficiency	Yes	27 (0.1)	26 (0.1)	1 (0.0)
Chronic heart failure	Yes	892 (2.1)	799 (2.0)	93 (2.8)
Chronic obstructive pulmonary disease	Yes	3456 (8.0)	3121 (7.8)	335 (10.0)
Dementia	Yes	445 (1.0)	421 (1.1)	24 (0.7)
Depression	Yes	3891 (9.0)	3636 (9.1)	255 (7.6)
Diabetes	Yes	5035 (11.6)	4564 (11.4)	471 (14.1)
Eating disorder	Yes	12 (0.0)	11 (0.0)	1 (0.0)
Inflammatory bowel disease	Yes	178 (0.4)	168 (0.4)	10 (0.3)
Multiple sclerosis	Yes	127 (0.3)	114 (0.3)	13 (0.4)
Renal failure	Yes	528 (1.2)	469 (1.2)	59 (1.8)
Rheumatoid arthritis	Yes	1306 (3.0)	1182 (3.0)	124 (3.7)
Thyroid disease	Yes	2118 (4.9)	1946 (4.9)	172 (5.1)
*During follow‐up*				
Number of weight measurement episodes	Median [IQR]	13 [6, 24]	14 [6, 25]	11 [5, 21]
Interval of weight measurements (days)	Median [IQR]	70.5 [38.0, 143.5]	73.0 [40.0, 147.0]	50.0 [29.0, 102.6]
Weight change (kg)	Median [IQR]	−0.38 [−5.03, 3.03]	−0.29 [−4.97, 3.14]	−1.13 [−5.66, 1.65]
Weight change episodes^a^	10% and over WG	2909 (0.4)	2901 (0.4)	8 (0.2)
	5 to <10% WG	27 050 (3.4)	26 949 (3.4)	101 (3.0)
	0 to <5% WG	386 856 (49.1)	385 370 (49.1)	1486 (44.3)
	>0 to 5% WL	340 150 (43.2)	338 592 (43.2)	1558 (46.5)
	>5 to 10% WL	27 493 (3.5)	27 322 (3.5)	171 (5.1)
	>10% and over WL	3182 (0.4)	3154 (0.4)	28 (0.8)
Bariatric procedure	Adjustable gastric band	13 (0.03)	13 (0.03)	0 (0.0)
	Roux‐en‐Y gastric bypass	238 (0.6)	223 (0.6)	15 (0.4)
	Sleeve gastrectomy	9 (0.02)	9 (0.02)	0 (0.0)
Exit reason	Disenrollment	4593 (10.6)	3492 (8.7)	1101 (32.8)
	Death	8852 (20.4)	8621 (21.6)	231 (6.9)
	Moved outside SEER	391 (0.9)	374 (0.9)	17 (0.5)
	DOB in 2014	29 466 (68.0)	27 463 (68.7)	2003 (59.8)
Total		43 302 (100.0)	39 950 (100.0)	3352 (100.0)

DOB, date of birth; kg, kilogram; SEER, Surveillance, Epidemiology, and End Results; WG, weight gain; WL, weight loss.

a The total number of weight change episodes far exceeds the total number of participants as each participant had multiple weight change episodes.

### Associations between weight change and cancer diagnosis

**Figure 2:**
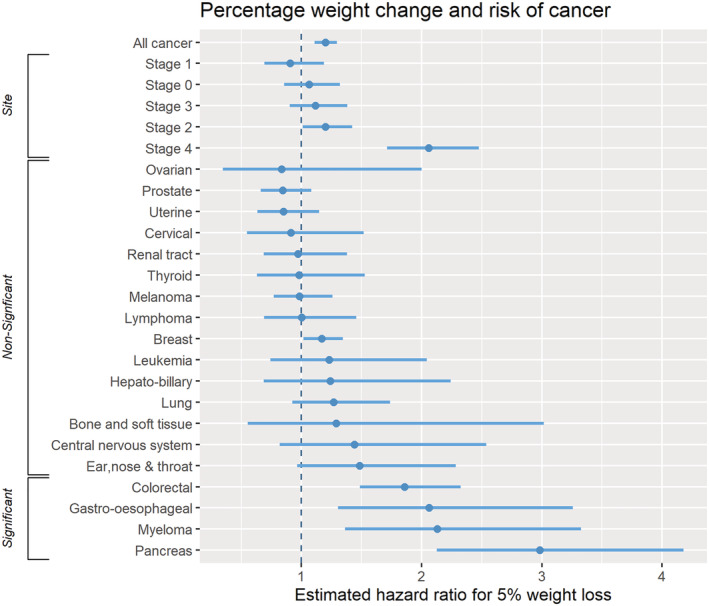
Minimally adjusted hazard ratios (95% confidence intervals) for 5% weight loss for all cancers and by cancer site and stage at diagnosis.

The overall HR (95% CI) for cancer diagnosis was 1.04 (1.02 to 1.05) for 1% weight loss, 1.20 (1.11 to 1.30) for 5%, and 1.44 (1.23 to 1.68) for 10% (*Table* 2, *Figure* 2, and Supporting Information, *Figure* S2). The interaction between percentage weight change and age at start of episode was found to be significant and was included in all models (Supporting Information, *Tables* S2 and S3). Of note, interactions between percentage weight change and baseline weight, percentage weight change and weight change interval, and between sex and percentage weight change, were not significant (Supporting Information, *Tables* S4 and S5). There were no significant differences in HRs generated by the minimally and fully adjusted models (Supporting Information, *Figure* S3).

**Table 2:** Adjusted hazard ratios for the association between weight loss and subsequent cancer diagnosis

Cancer site	*N* (%)	Hazard ratio (95% confidence interval)			
		1% weight loss	5% weight loss	10% weight loss	*P*‐value
All cancer	3352 (100.00)	1.04 (1.02 to 1.05)	1.20 (1.11 to 1.30)	1.44 (1.23 to 1.68)	0.000
Ear, nose & throat	108 (3.22)	1.08 (0.99 to 1.18)	1.48 (0.96 to 2.28)	2.20 (0.93 to 5.22)	0.073
Gastro‐oesophageal	76 (2.27)	1.16 (1.05 to 1.27)	2.06 (1.30 to 3.26)	4.25 (1.70 to 10.61)	0.002
Colorectal	292 (8.71)	1.13 (1.08 to 1.18)	1.86 (1.49 to 2.32)	3.46 (2.21 to 5.40)	0.000
Hepatobiliary	68 (2.03)	1.04 (0.93 to 1.18)	1.24 (0.69 to 2.24)	1.53 (0.47 to 5.02)	0.479
Pancreas	102 (3.04)	1.24 (1.16 to 1.33)	2.98 (2.13 to 4.18)	8.89 (4.52 to 17.48)	0.000
Lung	348 (10.38)	1.05 (0.98 to 1.12)	1.27 (0.93 to 1.74)	1.61 (0.86 to 3.02)	0.140
Bone and soft tissue	28 (0.84)	1.05 (0.89 to 1.25)	1.29 (0.55 to 3.02)	1.66 (0.30 to 9.10)	0.556
Melanoma	284 (8.47)	1.00 (0.95 to 1.05)	0.98 (0.77 to 1.26)	0.97 (0.59 to 1.58)	0.899
Breast	612 (18.26)	1.03 (1.00 to 1.06)	1.17 (1.02 to 1.34)	1.36 (1.03 to 1.80)	0.029
Cervical	48 (1.43)	0.98 (0.89 to 1.09)	0.91 (0.55 to 1.52)	0.83 (0.30 to 2.31)	0.721
Uterine	136 (4.06)	0.97 (0.91 to 1.03)	0.85 (0.63 to 1.15)	0.73 (0.40 to 1.31)	0.289
Ovarian	45 (1.34)	0.96 (0.81 to 1.15)	0.83 (0.35 to 2.00)	0.70 (0.12 to 4.00)	0.684
Prostate	360 (10.74)	0.97 (0.92 to 1.02)	0.85 (0.66 to 1.08)	0.71 (0.44 to 1.17)	0.181
Renal tract	247 (7.37)	0.99 (0.93 to 1.07)	0.97 (0.69 to 1.38)	0.94 (0.47 to 1.90)	0.873
Central nervous system	41 (1.22)	1.08 (0.96 to 1.20)	1.44 (0.82 to 2.54)	2.08 (0.67 to 6.44)	0.205
Thyroid	80 (2.39)	1.00 (0.91 to 1.09)	0.98 (0.63 to 1.53)	0.96 (0.40 to 2.33)	0.932
Lymphoma	166 (4.95)	1.00 (0.93 to 1.08)	1.00 (0.69 to 1.46)	1.01 (0.48 to 2.12)	0.989
Myeloma	56 (1.67)	1.16 (1.06 to 1.27)	2.13 (1.36 to 3.33)	4.54 (1.86 to 11.06)	0.001
Leukaemia	96 (2.86)	1.04 (0.94 to 1.15)	1.23 (0.74 to 2.04)	1.51 (0.55 to 4.17)	0.423
Other	132 (3.94)	1.12 (1.00 to 1.24)	1.73 (1.02 to 2.92)	2.99 (1.05 to 8.54)	0.040
Stage at diagnosis^a^					
Stage 0	352 (10.50)	1.01 (0.97 to 1.06)	1.06 (0.86 to 1.32)	1.13 (0.74 to 1.74)	0.572
Stage 1	938 (27.98)	0.99 (0.96 to 1.02)	0.95 (0.83 to 1.09)	0.91 (0.69 to 1.19)	0.477
Stage 2	622 (18.56)	1.04 (1.00 to 1.07)	1.20 (1.01 to 1.42)	1.44 (1.02 to 2.02)	0.039
Stage 3	409 (12.20)	1.02 (0.98 to 1.07)	1.12 (0.90 to 1.38)	1.25 (0.82 to 1.91)	0.308
Stage 4	503 (15.01)	1.16 (1.11 to 1.20)	2.06 (1.71 to 2.47)	4.24 (2.94 to 6.12)	0.000

‘Minimally adjusted’ Cox proportional hazards models included percentage weight change, age, sex, weight change interval, and tobacco use as time varying covariates.

a 15.75% of cancers did not have associated stage information.

### Cancer site and cancer stage

Associations between measured weight loss and the rate of cancer diagnosis varied by cancer site and cancer stage. Increased rates of pancreatic cancer, myeloma, gastro‐oesophageal, colorectal, and breast cancer diagnosis ranged from HR 1.17 (95% CI 1.02 to 1.34) for breast cancer to 2.98 (2.13 to 4.18) for pancreatic for 5% weight loss (*Table* 2, *Figure* 2). For bone and soft tissue, central nervous system, ear nose and throat, hepato‐biliary, leukaemia, and lung cancer the association was not statistically significant, but confidence intervals included clinically important increases in diagnosis rates (*Table* 2, *Figure* 2). For cervical, ovarian, prostate, and uterine cancer, hazard ratios were less than one, suggesting a lower rate of cancer diagnosis for measured weight loss, but the confidence intervals crossed one. The association between 5% weight loss and cancer diagnosis was generally stronger for more advanced cancer stages: stage I, HR 0.95 (95% CI 0.83 to 1.09); stage II, 1.20 (1.01 to 1.42); stage III, 1.12 (0.90 to 1.38); and stage IV, 2.06 (1.71 to 2.48).

### Absolute risk of cancer diagnosis

**Figure 3:**
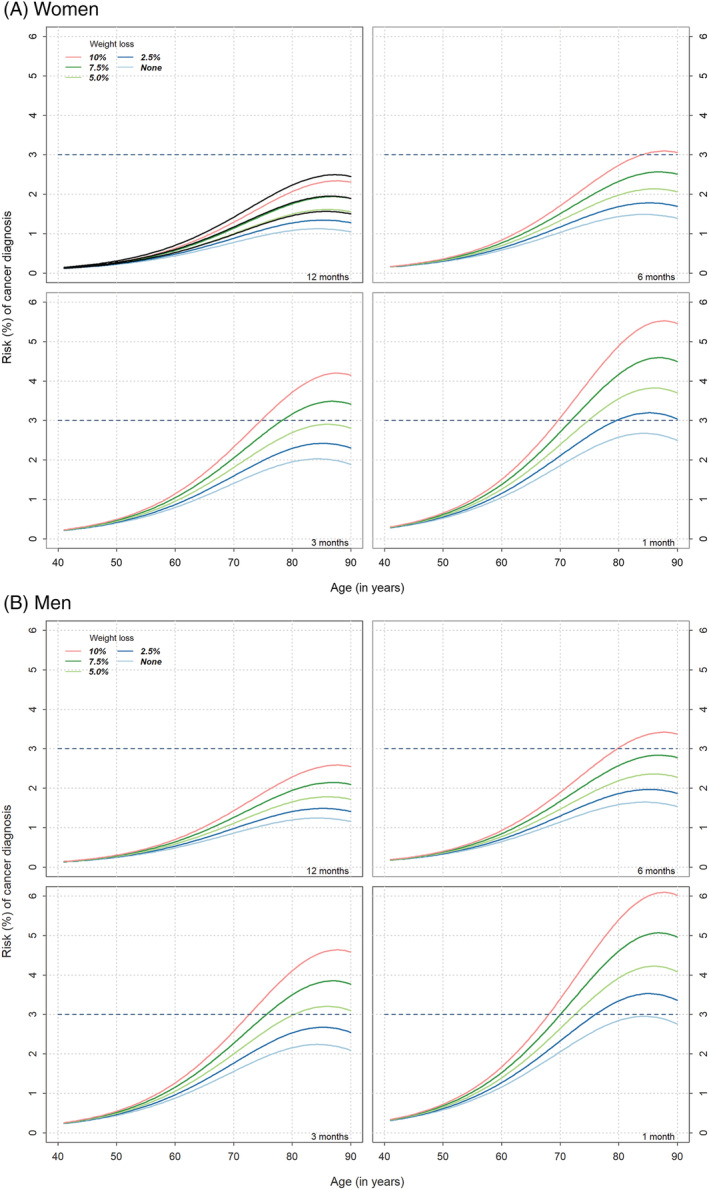
The risk of cancer diagnosis over a 6 month period by age and percentage weight loss over measurement intervals of 1, 3, 6, and 12 months in women (A ‐ upper four panels) and men (B‐ lower four panels). The dotted line represents 3% cancer risk, the threshold at which National Institute for Health and Care Excellence (NICE) recommends cancer investigation is initiated.

An increasing risk of cancer diagnosis was independently associated with a linear increase in the amount of weight loss, increasing age up to 85 years, and as the interval of weight measurement shortened below 12 months (Supporting Information, *Figure* S4). To illustrate the interplay of these covariates, *Figure* 3 demonstrates that for a 45‐year‐old women (non‐smoker) with no weight loss observed between two consultations within a 6 month period the risk of cancer diagnosis was 0.20%, increasing to 0.22% if there was 5% weight loss over the same period, and 0.27% if there was 10% weight loss (*Figure* 3A). The risk of cancer in a 70‐year‐old women with no weight loss observed between two consultations within a 6 month period was estimated to be 1.01%, increasing to 1.32% if there was 5% weight loss over the same period, and 1.90% if there was 10% weight loss. If the 70‐year old woman had had weight measured twice within 3 months the risk of cancer diagnosis was estimated to be 1.38% if there was no weight loss observed, a risk of 1.81% if 5% was observed and 2.60% if 10% weight loss occurred over 3 months. PPVs for cancer diagnosis increased with increasing age‐group up to 70–79 years then fell, were greater over 12 months compared to six, and in men compared with women (*Table* 3).

**Table 3:** Positive predictive values (PPV) for a cancer diagnosis within 6 and 12 months following a measured weight loss of ≥5%

	Age range	Men					Women				
		TP	FP	FN	TN	PPV % (95% CI)	TP	FP	FN	TN	PPV % (95% CI)
6 months											
	40 to 49	0	126	22	2770	0.00 (0.00 to 2.89)	2	211	43	3996	0.94 (0.11 to 3.35)
	50 to 59	7	259	100	5689	2.63 (1.06 to 5.35)	10	378	123	7000	2.58 (1.24 to 4.69)
	60 to 69	9	255	146	4822	3.41 (1.57 to 6.37)	10	278	126	5341	3.47 (1.68 to 6.29)
	70 to 79	7	117	141	2462	5.65 (2.30 to 11.29)	10	144	129	3074	6.49 (3.16 to 11.62)
	80+	5	89	99	1905	5.32 (1.75 to 11.98)	13	185	123	3076	6.57 (3.54 to 10.97)
12 months											
	40 to 49	0	126	26	2766	0.00 (0.00 to 2.89)	3	210	61	3978	1.41 (0.29 to 4.06)
	50 to 59	9	257	124	5665	3.38 (1.56 to 6.33)	14	374	166	6957	3.61 (1.99 to 5.98)
	60 to 69	13	251	206	4762	4.92 (2.65 to 8.27)	16	272	189	5278	5.56 (3.21 to 8.87)
	70 to 79	10	114	181	2422	8.06 (3.94 to 14.33)	12	142	178	3025	7.79 (4.09 to 13.22)
	80+	7	87	141	1863	7.45 (3.05 to 14.74)	14	184	177	3022	7.07 (3.92 to 11.58)

FN, false negative; FP, false positive; TN, true negative; TP, true positive.

### Sensitivity analyses

The association between 5% weight loss and cancer risk [1.20 (95% CI 1.11 to 1.30)] was not materially affected in the sensitivity analyses we conducted: excluding people with a previous cancer diagnosis (1.20, 1.11 to 1.30), adjusting for consultation rate (1.20 (1.11 to 1.30), in a patient level analysis (1.17, 1.01 to 1.37), and after adding other symptoms into the model (1.20, 1.10 to 1.30).

## Discussion

### Summary

Reductions in weight measured routinely at primary care visits in a large US integrated health care system were positively associated with a subsequent diagnosis of five cancer sites and stages II and IV cancers. We found a linear increase in the risk of cancer diagnosis as the amount of weight lost increased, which was independent of patients' age, sex, baseline weight, weight measurement interval, and co‐morbidities. The risk of cancer diagnosis also increased with increasing age (up to 85 years) and as the measurement interval decreased (below a year).

### Strengths and limitations

Our dataset included routinely collected objective weight measurement data from a random sample of nearly 50 000 members of a healthcare system with minimal exclusions. A benefit to studying the association between weight change and cancer using routinely collected data is that our results are relevant to the setting in which the data were collected, without having imposed an unrealistic or artificial measurement schedule. We minimized data loss in the primary analysis by considering each interval between weight records to be an episode, meaning each patient was included in the analysis multiple times contributing multiple weight change estimates and follow‐up periods (totalling 782 650 intervals). Through linkage to the SEER registry, we identified 3352 cancers, and cancer stage was 84.2% complete. Estimates of association had narrow 95% confidence intervals for cancer overall, cancer stage overall, for smaller estimates of weight change, and for the larger cancer site groupings. Replication of our analysis in a larger database will improve confidence in our findings for rarer cancer sites, cancer stage (including within cancer sites), and for larger percentage changes in weight.

As we analysed a random sample of routinely collected retrospective observational EHR data from 50 000 patients cared for by a large US integrated health care system, our results require further validation in other clinical settings, populations and subgroups of interest with varying cancer prevalence. There may be competing explanations for the associations observed as the data reflect not only the health of the patients, but also patients' frequency and type of interactions with the healthcare system.^24^ Weight is measured as a routine observation during most primary care (including ER) visits in in the USA.^21^ A prospective trial with standardized weight measurement showed weight to fluctuate by approximately 0.4% from week to week across age‐group, by sex, geographical region, and body mass index. It is plausible that this normal fluctuation may be magnified with variable retrospective measurements.^23^ Although we have not formally investigated the reason for each weight record nor established the proportion of primary care encounters that have an associated weight record in this setting, it is likely that visit frequency is the most likely determinant of the frequency of weight recording for most intervals of weight measurement included in our analysis. Although we excluded patient time following bariatric surgery, we did not use prescription records or free text records to evidence intentional weight loss. In this respect, our estimates of association between weight loss and a subsequent diagnosis of cancer may be considered to be conservative.

### Comparison to previous literature

We are aware of no analogous analysis of routinely collected primary care EHR data (from the USA). An EHR based study of 390 cases of pancreatic ductal adenocarcinoma (PDAC) managed at an academic referral centre in the USA showed that PDAC was preceded by unintentional measured weight loss in most patients, even those diagnosed at an early stage.^20^ Most of the literature reporting the association between weight loss and a subsequent cancer diagnosis in primary care has been conducted using United Kingdom EHR data where weight is not recorded routinely and is often missing in the EHR.^25^ All but one English study identified in a recent systematic review used clinician coding to define weight loss.^12^ As a decision to code is associated with a clinician suspecting there is a clinical problem weight loss will remain uncoded in a patient for whom weight change has gone unnoticed or the GP does not suspect serious illness. Although GP coding has been shown to represent a mean loss of ≥5% in a 6 month period, it is likely that there is under recording of smaller amounts of weight loss.^26^ In this study, weight was measured routinely and frequently allowing weight loss to be defined more precisely and irrespective of clinical concern.

Data from hospitalized patients most commonly associates cancer cachexia with pancreatic, gastro‐oesophageal, head and neck, lung, and colorectal cancer.^27^ Despite different definitions of weight loss in the US and UK primary care data, similar cancers were found to be associated with weight loss: pancreatic, gastro‐oesophageal, and colorectal cancer.^11^ There were also important differences: cancer of unknown primary, hepatobiliary, renal tract, lung, and lymphoma were clearly associated with weight loss in the UK data and not the USA; breast cancer and myeloma were associated in the USA and not the UK; and breast and prostate cancer were negatively associated with weight loss in the UK but not the US data. Stage II and IV cancers were associated with UWL in this US dataset and stage III (in men) and IV cancers in the English primary care literature.^11^

### Implications for research and practice

Published guidance for family physicians in the USA recommends that UWL of ≥5% over 6 to 12 months in adults >65 years should be investigated and the English National Institute for Health and Care Excellence (NICE) recommends urgent referral for the investigation of cancer in subgroups of patients presenting with UWL in primary care but provides no guidance on the extent or timing of weight loss.^13^, ^28^ We observed that the amount of weight loss is associated with a cancer diagnosis independently of measurement interval and report PPVs for UWL by age‐group. Our data support recommendations that focus on the percentage weight loss in relation to the patient's age rather than a focus on fixed intervals for weight change. Therefore, guidelines that necessitate an interval for weight change could contribute to missed opportunities to diagnose cancer.

Unexpected weight loss may prompt clinical enquiry and initial diagnostic testing in preference to immediate referral for definitive cancer investigation in age groups with lower PPVs. Given the many possible cancer types and non‐cancer diagnoses associated with UWL, guidance in the USA recommends a broad clinical evaluation including history, physical examination, laboratory tests, chest radiography, faecal occult blood testing, and possibly abdominal ultrasonography.^13^ Recent research from the UK has outlined which co‐occurring symptoms, signs, and abnormal blood test results could be used to prioritize cancer investigation in people with weight loss by increasing the PPV for cancer.^16^, ^17^ This research could be validated in non‐UK settings such as the USA where alternative approaches to weight measurement and weight loss recording exist, where the spectrum of patients consulting primary care is different, and in systems where similar blood tests are used with differing degrees of missingness. Broadening the differential diagnosis from cancer to the other serious diseases associated with weight loss could increase the yield of investigation further. Research is required to characterize which serious diseases should be included in the differential diagnosis based on the amount of weight lost and the patient's age and sex.

The differences in stage at diagnosis between the USA and UK suggest that there may be an opportunity to detect cancer at an earlier stage, when curative treatment is more likely, by introducing regular weight monitoring in primary care settings where weight measurement is not currently routine. The optimal frequency of testing remains an open question. We observed that shorter intervals of weight measurement were associated with a higher risk of cancer, independent of the percentage of weight change. This may suggest that it is desirable to measure a patient's weight more frequently in order to detect cancer earlier. However, visit frequency is driven by incident symptoms of ill health (including unexpected weight loss), pre‐existing co‐morbidity, and planned follow‐up visits, and people with more frequent measurements are likely to be a sicker subset of the population. Such confounding makes the interpretation of our findings challenging in the absence of an indicator to note the reason for each weight measurement. More frequent testing may also lead to an increase in false alarms triggering further testing if the current approach of percentage weight change between serial measurements is used. Ultimately, it will be necessary to rigorously evaluate the association between using changes in weight as triggers for clinical investigation and the risk of cancer mortality.

An alternative approach might be to introduce more sophisticated methods to detect clinically relevant weight change. Home monitoring of weight using smart technology, for example scales linked to mobile devices that integrate with the EHR, could vastly increase the number of weight measurements available to clinicians and researchers. Without the necessity for the patient to interact with healthcare, these (artificial intelligence) assisted technologies introduce the possibility of developing personalized weight monitoring schedules incorporating shorter measurement intervals not associated with illness or consultation, with potential benefits to chronic disease management as well as serious disease diagnosis. Alternatively, simple alerts could be implemented in the EHR to trigger when a chosen % weight of loss occurs over a specified time period, or more simply to calculate the % change in weight since the last weight measurement. However, pragmatic studies are required before implementing remote monitoring as methods development is required to identify the most appropriate approach to monitoring unstructured weight measurement data to flag changes that should prompt a clinician to suspect underlying cancer. Any approach must incorporate the underlying weight trajectory and be sensitive enough to trigger further investigation for cancer without triggering too many false alarms. The effectiveness of such approach would ultimately need to be assessed by comparing the risk of cancer mortality in patients who do versus do not undergo home monitoring.

## Conclusions

Weight loss is a sign of undiagnosed cancer in primary care regardless of the interval over which it occurs. Guidelines should resist giving an arbitrary cut‐off for the interval of weight loss and focus instead on the percentage of weight loss and the patient's age. Randomized controlled trials or high‐quality observational studies are needed to evaluate the cancer mortality benefits of using weight change in guidelines for cancer diagnostic workup.

## Funding

The National Institute for Health Research (UK) supported this study. The funders had no role in study design, data collection and analysis, decision to publish, or preparation of the manuscript. BDN was supported by National Institute for Health Research (NIHR) Doctoral Research Fellowship number (DRF‐2015‐08‐18) and an NIHR Academic Clinical Lectureship. FDRH acknowledges part‐funding from the NIHR Oxford Medtech and In‐Vitro Diagnostics Cooperative (MIC), the NIHR Oxford and Thames Valley Applied Research Collaboration (ARC). FDRH and JO acknowledge part‐funding from the NIHR Oxford Biomedical Research Centre (BRC). JM acknowledges part‐funding from the ARC, during the conduct of the study; and occasionally receives expenses for teaching Evidence‐Based Medicine. The views expressed are those of the authors and not necessarily those of the NHS, the NIHR, National Institutes of Health or the Centers for Disease Control and Prevention.

## Conflict of interest

None declared.

## Supporting information


**Figure S1:** Intervals of analysis
**Figure S2:** Minimally adjusted hazard ratios (95% confidence intervals) for 10% weight loss for all cancers and by cancer site and stage at diagnosis.
**Figure S3:** Fully and minimally adjusted hazard ratios for a 1% increase in percentage weight change for all cancers, by cancer site and cancer stage. Solid lines represent the minimally adjusted model estimates and confidence intervals. Dashed lines the corresponding estimates for the fully adjusted model.
**Figure S4:** Hazard ratio of cancer diagnosis for % weight change, age, and interval of weight measurement when modelled as splines after adjustment for all other covariates.
**Table S1:** Groupings of cancer site codes used in the analysis.
**Table S2:** Estimated hazard ratios and confidence intervals from a Cox proportional hazards model for all cancers combined and (minimally) adjusted for age, sex, measurement interval, baseline weight, tobacco use and age times weight loss interaction.
**Table S3:** Estimated hazard ratios and confidence intervals from a Cox proportional hazards model for all cancers combined and (fully) adjusted for age, sex, measurement interval, baseline weight, tobacco use and age*weight loss, past cancer, ethnicity, and comorbidity. Term
**Table S4:** All cancer model including interaction of baseline weight and percentage weight loss
**Table S5:** All cancer model including interaction of interval of weight loss and weight loss.
