# Supplementary material for: Measured weight loss as a precursor to cancer diagnosis: retrospective cohort analysis of 43 302 primary care patients
Source: J Cachexia Sarcopenia Muscle. 2022 Jul 28;13(5):2492–503. doi: 10.1002/jcsm.13051 (PMC9530580; doi:10.1002/jcsm.13051)
Supplement: Supplementary file 1 — Figure S1: Intervals of analysis Figure S2: Minimally adjusted hazard ratios (95% confidence intervals) for 10% weight loss for all cancers and by cancer site and stage at diagnosis. Figure S3: Fully and minimally adjusted hazard ratios for a 1% increase in percentage weight change for all cancers, by cancer site and cancer stage. Solid lines represent the minimally adjusted model estimates and confidence intervals. Dashed lines the corresponding estimates for the fully adjusted model. Figure S4: Hazard ratio of cancer diagnosis for % weight change, age, and interval of weight measurement when modelled as splines after adjustment for all other covariates. Table S1: Groupings of cancer site codes used in the analysis. Table S2: Estimated hazard ratios and confidence intervals from a Cox proportional hazards model for all cancers combined and (minimally) adjusted for age, sex, measurement interval, baseline weight, tobacco use and age times weight loss interaction. Table S3: Estimated hazard ratios and confidence intervals from a Cox proportional hazards model for all cancers combined and (fully) adjusted for age, sex, measurement interval, baseline weight, tobacco use and age*weight loss, past cancer, ethnicity, and comorbidity. Term Table S4: All cancer model including interaction of baseline weight and percentage weight loss Table S5: All cancer model including interaction of interval of weight loss and weight loss. [file JCSM-13-2492-s001.docx]

**Measured weight loss as a precursor to cancer diagnosis: retrospective cohort analysis of 43,302 primary care patients**

**Journal of Cachexia, Sarcopenia and Muscle**

Authors: Brian D Nicholson^1^, Matthew J Thompson^2^, FD Richard Hobbs^1^, Matthew Nguyen^3^, Julie McLellan^1^, Beverly Green^3^, Jessica Chubak^3^, Jason L Oke^1^

Affiliations

^1^Nuffield Department of Primary Care Health Sciences, University of Oxford, UK OX2 6GG

^2^Department of Family Medicine, University of Washington

^3^Kaiser Permanente Washington Health Research Institute

E-mail: brian.nicholson@phc.ox.ac.uk

**Online Supplementary Material**

**Figure S1: Intervals of analysis**
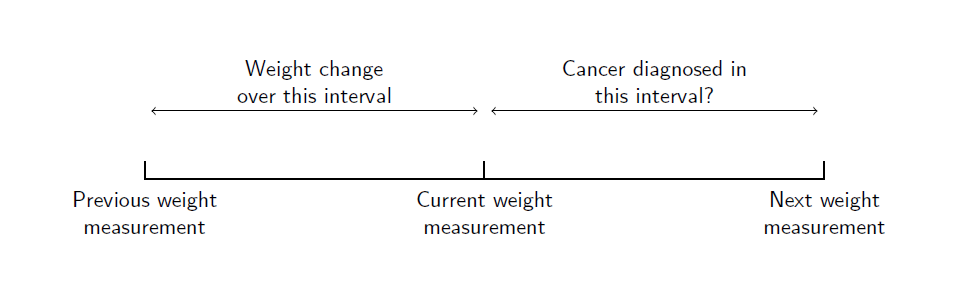


**Figure S2:** **Minimally adjusted hazard ratios (95% confidence intervals) for 10% weight loss for all cancers and by cancer site and stage at diagnosis.**


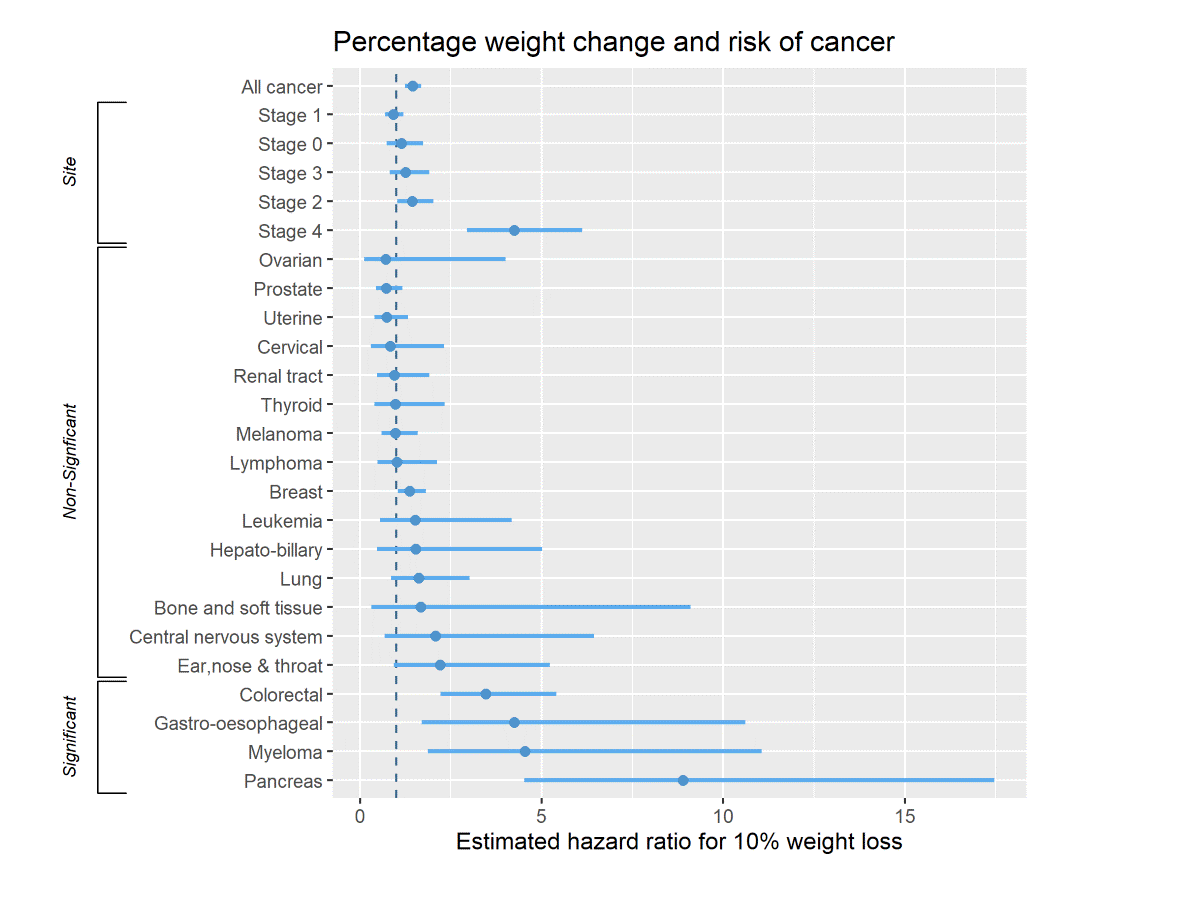


**Figure S3:** Fully and minimally adjusted hazard ratios for a 1% increase in percentage weight change for all cancers, by cancer site and cancer stage. Solid lines represent the minimally adjusted model estimates and confidence intervals. Dashed lines the corresponding estimates for the fully adjusted model.


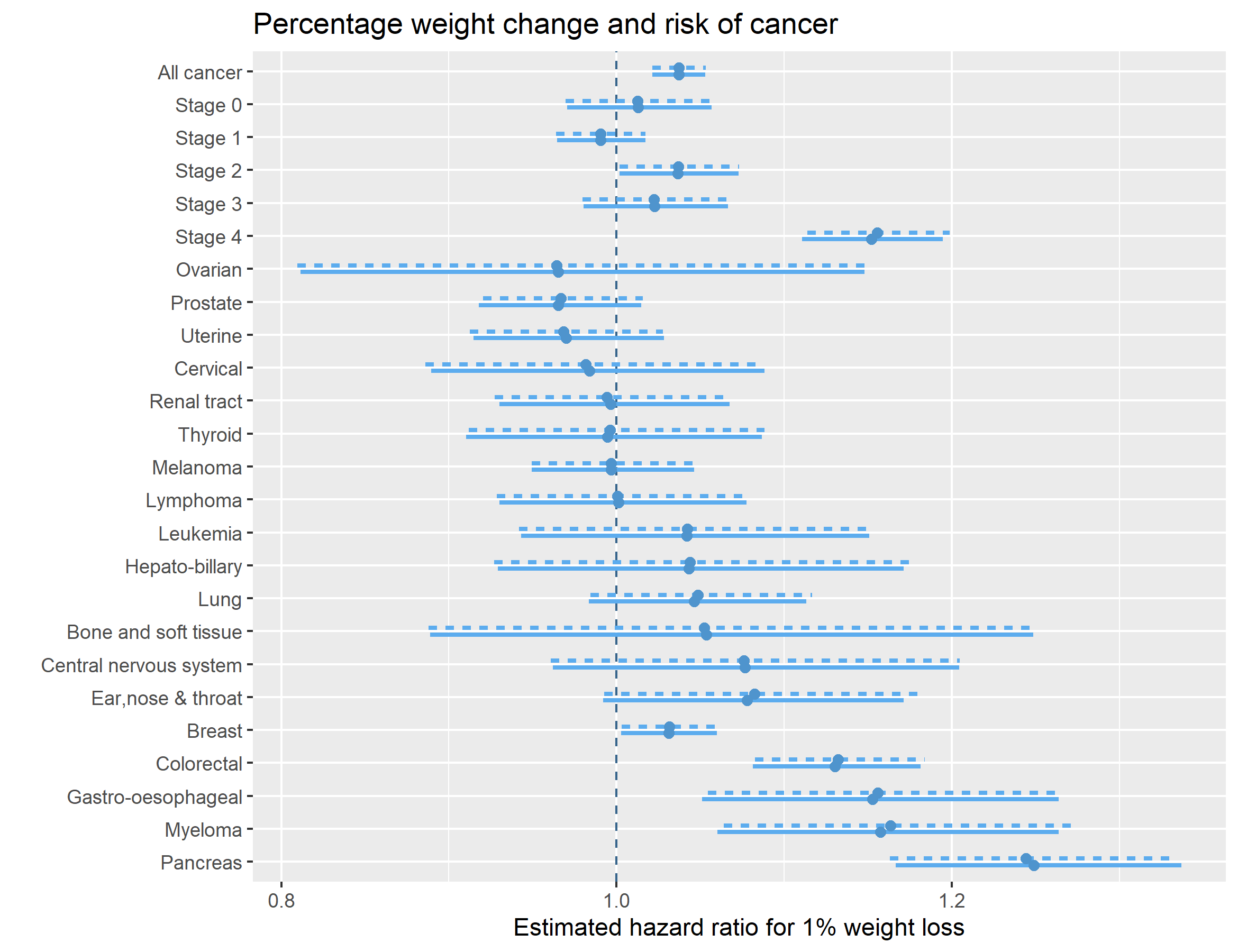


**Figure S4: Hazard ratio of cancer diagnosis for % weight change, age, and interval of weight measurement when modelled as splines after adjustment for all other covariates.**

The left-hand plot shows that as the amount of weight loss increases there is an independent linear increase in cancer diagnosis. The middle plot shows that increasing age above 60 years is independently associated with an increasing risk of cancer diagnosis up to age 84.3 years thereafter the risk returns to the risk at age 60 years as age increases. The right-hand plot shows that the shorter the time interval of measurement below 415 days (the greater the risk of cancer. The model included age, gender, baseline weight, percentage weight change, interval of weight measurement, tobacco use, and the interaction between age and percentage weight change. Confidence bands are based on pointwise standard errors.


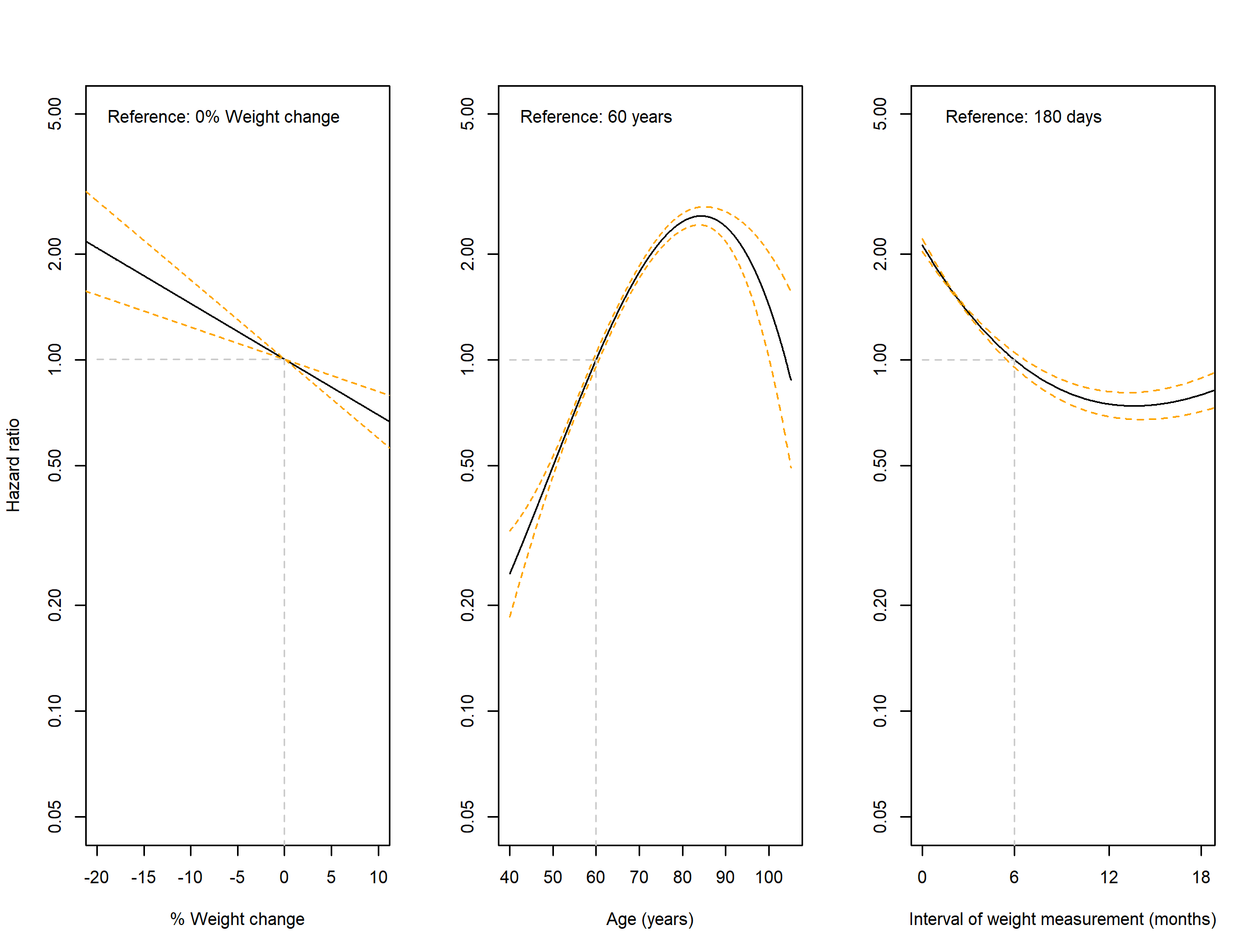


**Table S1:** Groupings of cancer site codes used in the analysis.

| Site | Code | Grouping |
| --- | --- | --- |
| Lip | 20010 | Ear, nose & throat |
| Tongue | 20020 |  |
| Salivary Gland | 20030 |  |
| Floor of Mouth | 20040 |  |
| Gum and Other Mouth | 20050 |  |
| Nasopharynx | 20060 |  |
| Tonsil | 20070 |  |
| Hypopharynx | 20090 |  |
| Nose, Nasal Cavity and Middle Ear | 22010 |  |
| Larynx | 22020 |  |
| Esophagus | 21010 | Gastro-oesophageal |
| Stomach | 21020 |  |
| Small Intestine | 21030 | Colorectal |
| Cecum | 21041 |  |
| Appendix | 21042 |  |
| Ascending Colon | 21043 |  |
| Hepatic Flexure | 21044 |  |
| Transverse Colon | 21045 |  |
| Splenic Flexure | 21046 |  |
| Descending Colon | 21047 |  |
| Sigmoid Colon | 21048 |  |
| Large Intestine, NOS | 21049 |  |
| Rectosigmoid Junction | 21051 |  |
| Rectum | 21052 |  |
| Anus, Anal Canal and Anorectum | 21060 |  |
| Liver | 21071 | Hepato-biliary |
| Intrahepatic Bile Duct | 21072 |  |
| Gallbladder | 21080 |  |
| Other Biliary | 21090 |  |
| Pancreas | 21100 | Pancreas |
| Lung and Bronchus | 22030 | Lung |
| Trachea, Mediastinum and Other Respiratory Organs | 22060 |  |
| Mesothelioma | 36010 |  |
| Retroperitoneum | 21110 | Bone and soft tissue |
| Peritoneum, Omentum and Mesentery | 21120 |  |
| Bones and Joints | 23000 |  |
| Soft Tissue including Heart | 24000 |  |
| Kaposi Sarcoma | 36020 |  |
| Melanoma of the Skin | 25010 | Melanoma |
| Other Non-Epithelial Skin | 25020 | N/A |
| Breast | 26000 | Breast |
| Cervix Uteri | 27010 | Cervical |
| Corpus Uteri | 27020 | Uterine |
| Uterus, NOS | 27030 |  |
| Ovary | 27040 | Ovarian |
| Vagina | 27050 | Vulvovaginal |
| Vulva | 27060 |  |
| Other Female Genital Organs | 27070 |  |
| Prostate | 28010 | Prostate |
| Testis | 28020 | Testes or Penis |
| Penis | 28030 |  |
| Other Male Genital Organs | 28040 |  |
| Urinary Bladder | 29010 | Renal tract |
| Kidney and Renal Pelvis | 29020 |  |
| Ureter | 29030 |  |
| Other Urinary Organs | 29040 |  |
| Eye and Orbit | 30000 | Central nervous system |
| Brain | 31010 |  |
| Cranial Nerves Other Nervous System | 31040 |  |
| Thyroid | 32010 | Thyroid |
| Hodgkin - Nodal | 33011 | Lymphoma |
| NHL - Nodal | 33041 |  |
| NHL - Extranodal | 33042 |  |
| Myeloma | 34000 | Myeloma |
| Acute Lymphocytic Leukemia | 35011 | Leukemia |
| Chronic Lymphocytic Leukemia | 35012 |  |
| Other Lymphocytic Leukemia | 35013 |  |
| Acute Myeloid Leukemia | 35021 |  |
| Chronic Myeloid Leukemia | 35022 |  |
| Other Myeloid/Monocytic Leukemia | 35023 |  |
| Aleukemic, subleukemic and NOS | 35043 |  |
| Other Digestive Organs | 21130 | Other |
| Miscellaneous | 37000 |  |
|  |  |  |

**Table S2:** Estimated hazard ratios and confidence intervals from a Cox proportional hazards model for all cancers combined and (minimally) adjusted for age, sex, measurement interval, baseline weight, tobacco use and age times weight loss interaction.

| Term | Level | Est. Hazard Ratio | Lower 95% CI | Upper 95% CI | p.value |
| --- | --- | --- | --- | --- | --- |
| Weight loss | % | 1.04 | 1.02 | 1.05 | 0.00 |
| Age | spline (1) | 4.26 | 1.98 | 9.20 | 0.00 |
|  | spline (2) | 40.36 | 25.03 | 65.07 | 0.00 |
|  | spline (3) | 3.57 | 1.65 | 7.70 | 0.00 |
| Sex (ref. female) | Male | 1.11 | 1.03 | 1.19 | 0.01 |
| Measurement Interval | spline (1) | 0.00 | 0.00 | 0.01 | 0.00 |
|  | spline (2) | 1,408,458.13 | 53,093.64 | 37,363,312.07 | 0.00 |
|  | spline (3) | 0.00 | 0.00 | 0.00 | 0.00 |
| Baseline Weight | Kg | 1.03 | 1.01 | 1.04 | 0.01 |
| Tobacco use (ref. current) | Missing | 0.77 | 0.66 | 0.89 | 0.00 |
|  | Other | 0.75 | 0.64 | 0.88 | 0.00 |
| Age * Weight loss |  | 1.00 | 1.00 | 1.00 | 0.00 |

**Table S3:** Estimated hazard ratios and confidence intervals from a Cox proportional hazards model for all cancers combined and (fully) adjusted for age, sex, measurement interval, baseline weight, tobacco use and age*weight loss, past cancer, ethnicity, and comorbidity. Term

|  | Level | Est. Hazard Ratio | Lower 95% CI | Upper 95% CI | p.value |
| --- | --- | --- | --- | --- | --- |
| Weight loss (%) | % | 1.04 | 1.02 | 1.05 | 0.00 |
| Age | spline (1) | 3.98 | 1.84 | 8.60 | 0.00 |
|  | spline (2) | 27.25 | 16.80 | 44.20 | 0.00 |
|  | spline (3) | 2.34 | 1.07 | 5.09 | 0.03 |
| Sex (ref. female) | Male | 1.17 | 1.08 | 1.26 | 0.00 |
| Interval | spline (1) | 0.01 | 0.00 | 0.01 | 0.00 |
|  | spline (2) | 1,039,232.43 | 40,091.99 | 26,938,148.52 | 0.00 |
|  | spline (3) | 0.00 | 0.00 | 0.00 | 0.00 |
| Baseline Weight (Kg) | Kg | 1.01 | 0.99 | 1.02 | 0.61 |
| Tobacco use (ref. current) | Missing | 0.81 | 0.69 | 0.94 | 0.01 |
|  | Other | 0.78 | 0.66 | 0.91 | 0.00 |
| Age * Weight loss |  | 1.00 | 1.00 | 1.00 | 0.00 |
| Past Cancer (ref. no) | Yes | 1.35 | 1.23 | 1.48 | 0.00 |
| Ethnicity (ref. white) | Non-White | 0.60 | 0.54 | 0.67 | 0.00 |
| Dementia (ref. no) | Yes | 0.91 | 0.78 | 1.07 | 0.25 |
| Depression (ref. no) | Yes | 0.97 | 0.89 | 1.05 | 0.45 |
| Diabetes (ref. no) | Yes | 1.00 | 0.92 | 1.10 | 0.94 |
| COPD (ref. no) | Yes | 1.21 | 1.11 | 1.31 | 0.00 |
| Heart Failure (ref. no) | Yes | 1.12 | 0.99 | 1.26 | 0.07 |
| Thyroid dysfunction (ref. no) | Yes | 1.02 | 0.92 | 1.13 | 0.71 |
| Inflammatory Bowel Disease (ref. no) | Yes | 0.82 | 0.59 | 1.14 | 0.23 |
| Rheumatoid Arthritis (ref. no) | Yes | 1.06 | 0.96 | 1.17 | 0.22 |
| Renal Function (ref. no) | Yes | 1.19 | 1.07 | 1.33 | 0.00 |

**Table S4:** All cancer model including interaction of baseline weight and percentage weight loss

| Term | Level | Est. Hazard Ratio | Lower 95% CI | Upper 95% CI | p.value |
| --- | --- | --- | --- | --- | --- |
| Weight loss | % | 1.03 | 1.02 | 1.05 | 0.00 |
| Age | spline (1) | 4.27 | 1.98 | 9.22 | 0.00 |
|  | spline (2) | 40.24 | 24.96 | 64.89 | 0.00 |
|  | spline (3) | 3.57 | 1.65 | 7.70 | 0.00 |
| Sex (ref. female) | Male | 0.00 | 0.00 | 0.01 | 0.00 |
| Measurement Interval | spline (1) | 1,431,925.77 | 53,946.48 | 38,008,249.52 | 0.00 |
|  | spline (2) | 0.00 | 0.00 | 0.00 | 0.00 |
|  | spline (3) | 1.11 | 1.03 | 1.19 | 0.01 |
| Baseline Weight | Kg | 1.02 | 1.01 | 1.04 | 0.01 |
| Tobacco use (ref. current) | Missing | 0.77 | 0.66 | 0.89 | 0.00 |
|  | Other | 0.75 | 0.64 | 0.88 | 0.00 |
| Age * Weight loss |  | 1.00 | 1.00 | 1.00 | 0.00 |
| Baseline weight * Weight loss |  | 1.00 | 1.00 | 1.01 | 0.24 |

**Table S5:** All cancer model including interaction of interval of weight loss and weight loss.

| Term | Level | Est. Hazard Ratio | Lower 95% CI | Upper 95% CI | p.value |
| --- | --- | --- | --- | --- | --- |
| Weight loss | % | 1.04 | 1.02 | 1.06 | 0.00 |
| Age | spline (1) | 4.26 | 1.98 | 9.20 | 0.00 |
|  | spline (2) | 40.33 | 25.01 | 65.03 | 0.00 |
|  | spline (3) | 3.56 | 1.65 | 7.70 | 0.00 |
| Sex (ref. female) | Male | 0.00 | 0.00 | 0.01 | 0.00 |
| Measurement Interval | spline (1) | 1,381,961.67 | 51,892.44 | 36,803,398.61 | 0.00 |
|  | spline (2) | 0.00 | 0.00 | 0.00 | 0.00 |
|  | spline (3) | 1.11 | 1.03 | 1.19 | 0.01 |
| Baseline Weight | Kg | 1.03 | 1.01 | 1.04 | 0.01 |
| Tobacco use (ref. current) | Missing | 0.77 | 0.66 | 0.89 | 0.00 |
|  | Other | 0.75 | 0.64 | 0.88 | 0.00 |
| Age * Weight loss |  | 1.00 | 1.00 | 1.00 | 0.00 |
| Measurement Interval * Weight loss |  | 1.00 | 1.00 | 1.00 | 0.83 |
